# Supplementary material for: A reservoir bubble point pressure prediction model using the Adaptive Neuro-Fuzzy Inference System (ANFIS) technique with trend analysis
Source: PLoS One. 2022 Aug 11;17(8):e0272790. doi: 10.1371/journal.pone.0272790 (PMC9371345; doi:10.1371/journal.pone.0272790)
Supplement: S1 Appendix — (PDF) [file pone.0272790.s001.pdf]

Supporting information

APPENDIX

Coefficient of determination (R^2)

The following equation determines the R^2:

$$R^2 = \left(\frac{n\sum xy - (\sum x)(\sum y)}{\sqrt{n(\sum x^2) - (\sum x)^2}\sqrt{n(\sum y^2) - (\sum y)^2}}\right)^2 \quad (1)$$

Relative deviation error

The relative deviation error (E_i) is given as follow:

$$E_i = \frac{Predicted\ Pb - measured\ Pb}{measured\ Pb} \quad (2)$$

i = 1, 2, 3, ..., n.

Average Percent Relative Error (APRE)

APRE is calculated from equation 3:

$$E_r = \left(\frac{1}{n}\right)\sum_{i=1}^{n} E_i \times 100 \quad (3)$$

Average Absolute Percentage Relative Error (AAPRE)

AAPRE can be obtained from the following equation:

$$E_a = \left(\frac{1}{n}\right)\sum_{i=1}^{n} |E_i| \times 100 \quad (4)$$

Correlation coefficient (R)

R is estimated from the following equation:

$$R = \sqrt{1 - \frac{\sum_{i=1}^{n}[measured\ Pb - predicted\ Pb]}{\sum_{i=1}^{n}[measured\ Pb - \underline{\Delta}Pb\ ]}} \tag{5}$$

Where:

$\underline{\Delta}Pb = \frac{1}{n}\sum_{i=1}^{N}[measured\ Pb]_i$

Standard deviation (SD)

SD is found from equation 6:

$$SD = \sqrt{\sum_{i=1}^{n} \frac{[\underline{x}_{errors} - x_{i\ errors}]^2}{n-1}} \tag{6}$$

Root Mean Square Error (RMSE)

RMSE is determined by the following:

$$RMSE = \sqrt{[\frac{1}{n}\sum_{i=1}^{n} E_i^2]} \tag{7}$$
